# A randomized, placebo-controlled, double-blinded, single-centre, phase IV trial to assess the efficacy and safety of OM-85 in children suffering from recurrent respiratory tract infections

**DOI:** 10.1186/s12967-019-2040-y

**Published:** 2019-08-23

**Authors:** Susanna Esposito, Sonia Bianchini, Samantha Bosis, Claudia Tagliabue, Ilaria Coro, Alberto Argentiero, Nicola Principi

**Affiliations:** 1 Pediatric Clinic, Department of Surgical and Biomedical Sciences, Università degli Studi di Perugia, Piazza Menghini 1, 06129 Perugia, Italy; 2 Pediatric Highly Intensive Care Unit, Department of Pathophysiology and Transplantation, Università degli Studi di Milano, Fondazione IRCCS Ca’ Granda Ospedale Maggiore Policlinico, Milan, Italy; 3 Università degli Studi di Milano, Milan, Italy

**Keywords:** Acute otitis media, Bacterial lysate, Common cold, OM-85, Recurrent respiratory tract infection

## Abstract

**Background:**

Over many years, OM-85, a lysate of 21 common bacterial respiratory pathogens, has been demonstrated to prevent respiratory recurrences in children. However, further studies are needed to explore the true importance of OM-85 in the prevention of respiratory tract infections (RTIs) in children. This study was planned to further contribute to the evaluation of the role played by OM-85 in prevention of recurrent RTIs in children.

**Methods:**

This study was a randomized (3:3:1), placebo-controlled, double-blind, single-centre, phase IV trial carried out in Italy to assess the efficacy of OM-85 (Broncho-Vaxom^®^; Vifor Pharma; Meyrin 2/Geneva, Switzerland) in reducing the number of new RTI episodes in 288 children aged 1 to 6 years with a history of recurrent RTIs and to compare the efficacy of the standard 3-month regimen with that of administration of OM-85 for 6 months during a 6-month study period.

**Results:**

The number of RTIs and of children who experienced at least one RTI were significantly lower among patients receiving OM-85 for 3 months than among those given placebo (33% vs 65.1%, p < 0.0001). Differences were statistically significant for upper RTIs (i.e., common cold/viral pharyngitis and acute otitis media; p < 0.0001 and p = 0.006, respectively). Days of absence from day-care for children and working days lost by parents were significantly lower in the group with children treated with OM-85 for 3 months than in the placebo group (p = 0.007 and p = 0.004, respectively). No difference was seen between children who received OM-85 for 3 and those who received OM-85 for 6 months. The prevalence of atopy as well as the history of recurrent wheezing and age of the study child did not influence the results. Benefit was maximally evident among children with a history of frequent recurrences. OM-85 was well tolerated and safe, even in children who received an influenza vaccination.

**Conclusions:**

The use of OM-85 for 3 months in 3 series of 10 consecutive days each time reduces the risk of recurrent RTIs in children, with a favourable safety profile. The greater effect observed in children prone to several respiratory episodes than in non-prone children seems to indicate that this lysate should be administered especially to children with a proven high susceptibility to RTIs.

## Background

In otherwise healthy infants and young children, respiratory tract infections (RTIs) are extremely common. In most cases, they are frequently recurrent and, despite being generally mild and spontaneously resolving in a few days, they cause significant medical, social and economic problems for children, their families and society [1–3]. Immunological immaturity, genetic characteristics and environmental factors, such as exposure to air pollutants, admission to a day-care centre, lack of breastfeeding and sedentary lifestyle, are considered the most important factors promoting RTIs [3].

To reduce the total burden of RTIs, prevention of recurrences through elimination of environmental factors and systematic use of available vaccines are strongly suggested [4]. Unfortunately, total elimination of environmental factors is very difficult and, in some cases, almost impossible [5]. Vaccines are only partially effective. Polysaccharide pneumococcal conjugate vaccines protect from the infections of no more than 13 pneumococcal serotypes although more than 90 serotypes can cause disease [6]. Finally, influenza vaccines are effective in no more than 80% of vaccinated children, with significant reduction of efficacy when mismatch between circulating vital strains and strains included in the vaccine occurs [7]. To improve protection, boosting immune system activity has been suggested [8].

OM-85, a lysate of 21 common bacterial respiratory pathogens, has been considered a possible measure in this regard [9, 10]. In vitro and in experimental animals, OM-85 induces synergistic toll like receptor (TLR)-2/6- and TLR-9-dependent innate immunity [11]. It activates mucosal dendritic cells in gastrointestinal Peyer’s patches to stimulate the activity of both innate and adaptive immune system responses [12].

Antimicrobial peptides released, and macrophages are activated with increased secretion of proinflammatory cytokines and anti-viral chemokines (e.g., type I interferons). Th1/Th2 imbalance, characteristic of the early periods of life, is corrected. Finally, B cell cytokines are produced, leading to increased production of immunoglobulins [12]. In general, animals given OM-85 are protected from respiratory infections [13].

Over many years, it has been demonstrated that OM-85 can prevent respiratory recurrences in children. Some years ago, Schaad reported in a meta-analysis of 8 randomized controlled trials that children treated with OM-85 had significantly and consistently fewer cases of recurrent RTIs [14]. This finding was confirmed by a more recent meta-analysis of 53 randomized controlled trials involving 4851 paediatric patients [15]. However, it was concluded that further studies were needed to explore the true importance of OM-85 in the prevention of RTIs in children. This study was planned to further contribute to the evaluation of the role played by OM-85 in prevention of recurrent RTIs in children.

## Methods

### Study design

This study was a randomized (3:3:1), placebo-controlled, double-blind, single- centre, phase IV trial carried out in Italy between October 1, 2016, and March 31, 2017, to assess the efficacy of OM-85 (Broncho-Vaxom^®^; Vifor Pharma; Meyrin 2/Geneva, Switzerland) in reducing the number of new RTI episodes in children with a history of recurrent RTIs and to compare the efficacy of the standard 3-month regimen with that of administration of OM-85 for 6 months. The study was approved by the Ethics Committee of the Fondazione IRCCS Ca’ Granda Ospedale Maggiore Policlinico, Milan, Italy, and conducted in accordance with the standards of Good Clinical Practice for trials of medicinal products in humans. The participants’ parents or legal guardians gave their written informed consent before the children were enrolled.

### Study population

The study involved children aged 1 to 6 years with a history of recurrent RTIs defined as at least 6 documented episodes in the previous year. Patients had to be regularly followed in the outpatient section for respiratory disorders of the Pediatric Highly Intensive Care Unit of the Fondazione IRCCS Ca’ Granda Ospedale Maggiore Policlinico, Milan, Italy. The children were screened during August and the first 15 days of September 2016 by means of a physical examination and a careful review of their medical history to assure compliance with the inclusion and exclusion criteria. At the time of selection, children in whom malformations of the cardiovascular system and the respiratory tract; chronic diseases of the lung, kidney and liver; primary or secondary immunodeficiencies; cancer; malnutrition; and severe allergic manifestations such as asthma and severe allergic dermatitis have been previously documented were excluded. Children with a history of documented viral wheezing, i.e. those with wheezing always associated with a viral infection, were included. Patients who were given antibiotics or systemic, inhaled or oral steroids within 4 weeks before enrolment or were currently enrolled in or had completed any other investigational device or drug study were excluded.

Eligible children were re-examined in the last week of September 2016 to decide definitive enrolment in the study that was planned to last 6 months. No exclusion factors emerged, and children were randomized by means of a random number generator in a 3:3:1 ratio to be included in one of the following treatment groups. Group A included children receiving 3.5 mg of OM-85 per os once a day for the first 10 days of each the first 3 months and placebo with the same scheme of administration for the second 3 months. Group B included children receiving always placebo for the first 10 days of each of the 6 months of the study. Group C included children given 3.5 mg of OM-85 once a day for the first 10 days of each of the 6 months. OM-85 was administered through capsules that can be opened and the content poured and dissolved into a 10 mL drink (i.e., water, fruit juice, milk). Each capsule contained 3.5 mg of standardized lyophilized bacterial extracts of *Haemophilus influenzae, Streptococcus pneumoniae, Klebsiella pneumoniae, Klebsiella ozaenae, Staphylococcus aureus, Streptococcus pyogenes, Streptococcus viridans,* and *Moraxella catarrhalis*. The study was blinded by labelling identical bottles of OM-85 capsules and placebo capsules and only revealing the randomization codes to the staff at the data monitoring centre, who had no contact with the patients; similarly, the physicians involved in clinical monitoring were blinded to the treatment assignment. The parents of children included in Groups A and B were given 3 numbered bottles, each of which contained an amount of liquid needed for 10 days of treatment. The children included in Group C received 6 bottles, each with the amount of OM-85 needed for 10 days of therapy. A portion of these children on the day of enrolment, on the basis of parents’ decisions, were vaccinated with an inactivated trivalent influenza vaccine (Fluarix, GSK, Verona, Italy), as follows: 44/123 (35.8%) in Group A, 43/124 (34.6%) in Group B and 15/41 (36.5%) in Group C. Local and systemic adverse events in vaccinated children were monitored over the 14 days following vaccination, as previously described [16].

To monitor the incidence of new episodes of RTI after study entry, the parents were asked to return to the Center with their child for a control visit at 1, 3 and 6 months after enrolment, bringing the bottles of capsules used for the study. Moreover, they were given a diary in which all their child’s clinical problems occurred between visits and the daily administration of preparations were to be recorded. When not diagnosed during prefixed visits, respiratory diseases had to be confirmed by the paediatrician of the National Health System regularly following the child and systematically recorded in the diary. For all the visits, prescribed medication was recorded and reported in the diary. Moreover, the parents were also asked to report in a special section of the diary both the absence of the child from day-care and the consequences on other family members of the respiratory infections of the child, such as the number of working days lost by parents due to caring for their ill children. In all the cases, respiratory diseases were classified into groups on the basis of signs and/or symptoms using well-established criteria [17]. In the case of signs and symptoms of more than one disease, the children were entered in the more severe disease group. Acute otitis media was suggested by fever and otalgia and definitively established by tympanic membrane examination by means of pneumatic otoscopy. Community-acquired pneumonia was diagnosed on the basis of clinical signs and symptoms and confirmed by means of chest radiography [18]. Viral wheezing was diagnosed considering medical history and lung sounds auscultation.

Compliance with the study regimen was verified by examining the parents’ diaries and inspecting the returned medication bottles containing capsules at the end of each visit.

### Statistical analysis

A total of 288 patients were planned to be randomized. This sample size was calculated to demonstrate a mean difference of at least one RTI between the standard treatment group (3 months treatment) and that of the placebo group at 6 months with a power of 80% and a common standard deviation (SD) of 2.5 (alpha of 5%, two-sided). The second active treatment arm was considered exploratory and was not used for inferential comparison but was only descriptive. Randomization was stratified by documented atopy/allergy or recurrent wheezing.

Continuous variables were expressed as the mean values ± SDs and were analysed using a two-sided Student’s test if they were normally distributed (on the basis of the Shapiro–Wilk statistic) or a two-sided Wilcoxon rank-sum test if they were not; categorical variables were expressed as numbers and percentages and were analysed using contingency table analysis and a Chi squared or Fisher’s test, as appropriate. Multivariate analysis was performed to evaluate the impact of risk factors for recurrent RTIs in the study population. All the analyses were two-tailed, and p values of < 0.05 were considered significant.

## Results

Among the 288 enrolled children, 123 were part of Group A, 124 were part of Group B and 41 were part of Group C. A total of 246 children (85.4%; 100 [81.3%] in Group A, 109 [87.9%] in Group B, and 37 [90.2%] in Group C) completed the study. In most of the cases (35 out of 42, 83.3%), refusal of parents to continue the study after the first or second month following definitive enrolment was the reason for the study exit. In the remaining cases, the study was stopped because of the refusal of the child to accept the prescribed preparation.

In Table 1, demographic characteristics of the study patients are reported. As shown, the groups were comparable, as no statistically significant difference among the groups was detected with regard to sex, age, ethnic group, residence, parents’ education, passive smoking from parents, number of siblings, day-care attendance, prevalence of atopy and history of recurrent wheezing. Among children with recurrent wheezing, 3 in Group A (16.7%), 3 in Group B (14.3%), and one in Group C (16.7%) had a previous diagnosis of a single episode of community-acquired pneumonia; none of the other study patients suffered from community-acquired pneumonia before enrollment.

**Table 1:** Baseline characteristics of children enrolled in the trial, by randomization group

Characteristic	Group A treatment, 3 months (n = 123)		Group B placebo (n = 124)		Group C treatment, 6 months (n = 41)	
	N	%	n	%	n	%
Sex						
Male	72	58.5	81	65.3	26	63.4
Female	51	41.5	43	34.7	15	36.6
Age at baseline (years)						
1–2	63	51.2	46	37.1	16	39.0
3–4	41	33.3	50	40.3	12	29.3
5–6	19	15.5	28	22.6	13	31.7
Mean ± SD	3.6 ± 1.6		3.7 ± 1.5		3.8 ± 1.7	
Ethnic group						
Caucasian	104	84.5	102	82.3	36	87.8
Any other	19	15.5	22	17.7	5	12.2
Residence						
Milan City	59	48.0	72	58.1	26	63.4
Outside Milan	64	52.0	52	41.9	15	36.6
Education (parents)^a^						
Primary/secondary	9	7.3	9	7.4	4	9.8
High school degree (at least one parent)	60	48.8	43	35.2	14	34.1
University degree (at least one parent)	54	43.9	70	57.4	23	56.1
Passive smoking (from parents)						
No	81	66.4	89	71.8	26	63.4
Yes (at least one parent)	41	33.6	35	28.2	15	36.6
No. of siblings						
0	28	22.8	31	25.0	13	31.7
1	75	61.0	73	58.9	20	48.8
2+	20	16.3	20	16.1	8	19.5
Attending nursery/primary school						
No	18	14.6	9	7.3	5	12.2
Yes	105	85.4	115	92.7	36	87.8
History of atopy						
No	96	78.1	95	76.6	32	78.1
Yes	27	21.9	29	23.4	9	21.9
History of recurrent wheezing						
No	105	85.4	103	83.1	35	85.4
Yes	18	14.6	21	16.9	6	14.6

No statistically significant differences were detected between groups

^a^The sums may not add up to the total because of missing values

Table 2 reports the impact of OM-85 treatment during the 6 months of the study period. Comparison of Groups A and B shows that the number of RTI episodes and the number of children that experienced at least one episode of RTI during the study period were significantly lower among patients receiving OM-85 for 3 months than among those given placebo (33% vs 65.1%, p < 0.0001). Differences were statistically significant also for upper RTIs (i.e., common cold/viral pharyngitis and AOM; p < 0.0001 and p = 0.006, respectively). The incidences of streptococcal pharyngitis, AOM with otorrhea and wheezing were lower in children given OM-85 for 3 months, but the differences did not reach statistical significance. In the majority of the children with lower respiratory tract infection diagnosis of viral wheezing was made and all the patients who reported wheezing episodes had a previous history of recurrent wheezing. Community-acquired pneumonia was reported only in one patient in Group A, one in Group B and none in Group C. Bacterial lysate administration was associated with a relevant reduction of markers of socioeconomic impact associated with recurrent RTIs. None of the patients were hospitalized during the study period. Days of absence from day-care for children and working days lost by parents were significantly lower in Group A than in Group B children (p = 0.007 and p = 0.004, respectively). No difference in any of the studied parameters was seen between children who received OM-85 for 3 months and those given the lysate for 6 months. The prevalence of atopy as well as the history of recurrent wheezing and age of the study child did not influence the results. Analysis of respiratory infection occurrence during the study period indicates that differences between children receiving OM-85 and the placebo group increased progressively during the first 3 months of active therapy and remained substantially lower for the last three months, independent of the duration of OM-85 administration (Table 3). Table 4 shows the cumulated percentage of patients reporting ≥ 3 upper RTIs and ≥ 3 AOM episodes during the study period, showing that benefits were maximally evident in this sub-population.

**Table 2:** Acute respiratory infections during follow-up, according to the randomization group

	Group A treatment, 3 months (n = 100)	Group B placebo (n = 109)	Group C treatment, 6 months (n = 37)	*p* value A vs B	p-value A vs C	p-value C vs B
Respiratory infections (any)	n (%)	n (%)	n (%)			
No	67 (32.0)	38 (34.9)	26 (70.3)			
Yes	33 (33.0)	71 (65.1)	11 (29.7)	< 0.0001	0.87	0.0003
AOM (any)						
No	76 (76.0)	60 (55.1)	28 (75.7)			
Yes	24 (24.0)	49 (44.9)	9 (24.3)	0.002	0.85	0.04
Antibiotic prescription						
No	75 (75.0)	54 (49.5)	27 (73.0)			
Yes	25 (25.0)	55 (50.5)	10 (27.0)	0.0002	0.98	0.02
Type of infection						
URTI, mean ± SD	0.33 ± 0.61	0.65 ± 0.55	0.29 ± 0.28	< 0.0001	0.85	0.0007
Bacterial pharyngitis, mean ± SD	0.10 ± 0.55	0.14 ± 0.66	0.12 ± 0.49	0.88	0.91	0.93
AOM, mean ± SD	0.24 ± 0.41	0.78 ± 0.73	0.25 ± 0.63	0.006	0.82	0.03
Otorrhea, mean ± SD	0.16 ± 0.55	0.22 ± 0.64	0.19 ± 0.72	0.66	0.88	0.69
Wheezing, mean ± SD	0.30 ± 0.63	0.27 ± 0.49	0.14 ± 0.54	0.78	0.06	0.08
Socioeconomic impact						
Days of absence from day-care, mean ± SD	4.49 ± 1.10	5.10 ± 1.33	4.33 ± 1.09	0.007	0.79	0.003
Working days lost by parents, mean ± SD	1.76 ± 0.76	2.58 ± 0.73	1.88 ± 0.88	0.004	0.82	0.0004

AOM: Acute otitis media; SD: standard deviation; URTI: upper respiratory tract infection; LRTI: lower respiratory tract infection

**Table 3:** Mean monthly upper respiratory tract infection and acute otitis media rate in the study population, according to treatment group

Infection	Month					
	1	2	3	4	5	6
URTI						
Group A treatment 3 months (n = 100)						
Mean	0.37	0.31*	0.28*	0.27*	0.31*	0.33
SD	0.37	0.36	0.46	0.31	0.49	0.61
Group B placebo (n = 109)						
Mean	0.43	0.46	0.43	0.58	0.61	0.65
SD	0.49	0.58	0.52	0.46	0.61	0.55
Group C treatment 6 months (n = 37)						
Mean	0.39	0.33*	0.31*	0.28*	0.30*	0.29
SD	0.25	0.30	0.22	0.24	0.19	0.28
AOM						
Group A treatment 3 months (n = 100)						
Mean	0.37	0.29*	0.27*	0.24*	0.26*	0.24*
SD	0.44	0.55	0.40	0.53	0.44	0.41
Group B placebo (n = 109)						
Mean	0.46	0.52	0.49	0.56	0.66	0.78
SD	0.51	0.63	0.60	0.66	0.72	0.73
Group C treatment 6 months (n = 37)						
Mean	0.35	0.31*	0.28*	0.26*	0.27*	0.25*
SD	0.31	0.28	0.30	0.31	0.25	0.33

AOM: acute otitis media; URTI: upper respiratory tract infection

* p < 0.05 vs placebo; no other significant differences between the groups

**Table 4:** Cumulated percentage of patients reporting ≥ 3 upper respiratory tract infections and ≥ 3 acute otitis media episodes during the study period

Infection	Month					
	1 (%)	2 (%)	3 (%)	4 (%)	5 (%)	6 (%)
URTI						
Group A treatment 3 months	0	5	12*	14*	18*	21*
Group B placebo	0	7	22	31	40	52
Group C treatment 6 months	0	6	11*	15*	16*	17*
AOM						
Group A treatment 3 months	0	2	6*	13*	19*	21*
Group B placebo	0	4	15	24	36	44
Group C treatment 6 months	0	3	7*	12*	16*	19*

AOM: acute otitis media; URTI: upper respiratory tract infection

* p < 0.05 vs placebo; no other significant differences between the groups

OM-85 was well tolerated and safe. Compliance was appropriate, and treatment was never withdrawn due to severe adverse events. Two patients in Group A and 3 in Group B had transient diarrhoea during the first period of OM-85 administration, whereas one patient in Group C suffered from cough during the fourth month of treatment. Table 5 shows the safety of influenza vaccination during the 14 days following its administration in those children who received influenza vaccine. Local and systemic tolerability were good regardless of the study group, and no serious adverse events were reported.

**Table 5:** Safety of influenza vaccination during the 14 days following administration, by randomization group

Characteristic	Treatment, 3 months (n = 44)		Placebo (n = 43)		Treatment, 6 months (n = 15)	
	n	%	n	%	n	%
Local adverse events	3	6.8	3	6.9	1	6.7
Erythema	3	6.8	2	4.7	1	6.7
Swelling/induration	1	2.3	1	2.3	1	6.7
Systemic adverse events	4	9.0	4	9.3	2	13.3
Fever ≥ 38 °C	3	6.8	2	4.7	1	6.7
Irritability	4	9.0	3	6.9	1	6.7
Lack of appetite	3	6.8	2	4.7	1	6.7
Vomiting	1	2.3	0	0	0	0
At least one local or systemic adverse event	6	13.6	7	16.3	3	20.0
Required drugs for adverse events	3	6.8	4	9.3	1	6.7
Severe adverse events	0	0	0	0	0	0

## Discussion

This randomized, placebo-controlled, double-blinded, single-centre, phase IV trial showed: (1) the best scheme of administration of OM-85 (i.e., for 3 months in 3 series of 10 consecutive days each time), (2) that OM-85 can be effective in reducing recurrent RTIs; (3) that clinical benefit of OM-85 is particularly marked in children at high risk of RTIs.

Children without known severe underlying disease commonly suffer, particularly during the cold months, from several respiratory infections involving mainly the upper respiratory tract [1–3]. This causes several medical and socioeconomic problems that are only partially reduced by traditional preventive measures, such as elimination of environmental factors favouring respiratory infections and use of vaccines. Despite the limitation derived by the relatively small number of patients enrolled, this randomized, placebo-controlled study seems to indicate that administration of OM-85 for 3 months in 3 series of 10 days each time can be effective in reducing the risk of new infections in patients who have already experienced several RTIs, thus limiting the total burden of these diseases. The data collected with this study confirm what have been already suggested by some previous studies but give new information regarding the best schedule of OM-85 administration and the duration of bacterial lysate protective effect. We found that during the study period, at least one new episode of RTI was diagnosed in over 65% of children who were given placebo and only in approximately one-third of those who received OM-85. The clinical benefit of OM-85 was particularly marked in children at high risk of RTIs, as it was found that the number of patients who had suffered from ≥ 3 episodes of common cold or pharyngitis at the end of the study was approximately 50% among children given placebo and only 21% among those treated with OM-85. All these findings are similar to those that have been previously reported by Schaad et al. [19], although in this study, the reduction of recurrences was greater than that found by these previous authors. In this study, recurrences in treated children were reduced by approximately 50% in comparison to those in untreated subjects, whereas reduction in the active-treatment group of the Schaad et al. [19] study was limited to 16%. Differences in the characteristics of the enrolled patients, monitoring of infections, and duration of the study period may explain the different results. Impact of OM-85 was evidenced on all the common respiratory infections, including wheezing, although in this case, differences between treated children and controls were not statistically significant. This finding seems clinically important as in several cases repeated episodes of viral wheezing have been found associated with development of asthma in later age [20]. On the other hand, reduction of viral wheezing is not surprising as a significant impact on wheezing incidence after OM-85 administration had been already reported by Razi et al. [21]. These authors used OM-85 or placebo with a scheme of administration quite similar to that prescribed in this study in a group of children aged 1–6 years with a history of recurrent wheezing. They reported that treated patients had a 37.9% reduction in wheezing attacks compared with the group given placebo and that this positive effect was evident for several months after lysate withdrawn.

As evidenced by some previous studies [10, 22], reduction of RTIs was accompanied by significant further advantages. The total number of antibiotic courses prescribed to children receiving OM-85 was significantly lower. Taking into account the role of antibiotic overuse in favouring the emergence of antimicrobial antibiotic resistance and related problems [23–25], this finding can be considered a relevant additional benefit of OM-85 administration. Moreover, both children and parents had significant social advantages. Children lost a significantly lower number of day-care attendance days, and parents lost a lower number of working days, in the treatment group than in the placebo group, with obvious advantages, even from an economic point of view.

A new, interesting finding of this study regards the best scheme of administration of OM-85 and the duration of protection offered by this bacterial lysate. Children who received 3.5 mg of the lysate for the first 10 days of 3 consecutive months had the same mean number of RTIs as those who were continuously treated for 6 months with the same monthly scheme. This result seems to suggest that the immune stimulation obtained with administration of OM-85 for 3 months persists for at least three additional months and that longer treatment periods are useless. The period with the highest risk of RTIs in the northern hemisphere usually starts in October and persists until March. This fact means that in the countries sited in Europe, Asia and northern America administration of OM-85 in the last 3 months of the year, may be sufficient to protect RTI-prone children s until the end of the risk period. As recently demonstrated [10], repetition of OM-85 could be useful in subsequent season(s) if risk factors for respiratory recurrences persist.

Finally, this study confirms that, as previously reported [2, 14, 25], the safety profile and tolerability of OM-85 were high, and compliance was good. Concomitant administration of influenza vaccine did not influence safety results. This highlights what has been suggested by Esposito et al. [15], i.e. that both measures can be used simultaneously so favouring associated reduction in the risk of RTIs.

## Conclusion

This study shows for the first time the best scheme of administration of OM-85 and supports the benefit-risk profile and therefore the use of OM-85 to reduce the risk of recurrent RTIs in children. The greater effect in children prone to several respiratory episodes than in non-prone children seems to support the use of this immunotherapy especially to children with a proven high susceptibility to RTIs.

## Data Availability

The data and materials used are included in the manuscript.

## Abbreviations

AOM: acute otitis media
RTIs: respiratory tract infections
SD: standard deviation
